# Tumor Suppressor Function of CYLD in Nonmelanoma Skin Cancer

**DOI:** 10.1155/2011/614097

**Published:** 2011-12-17

**Authors:** K. C. Masoumi, Gina Shaw-Hallgren, Ramin Massoumi

**Affiliations:** Molecular Tumor Pathology, Department of Laboratory Medicine, Lund University, Skåne University Hospital, 205 02 Malmö, Sweden

## Abstract

Ubiquitin and ubiquitin-related proteins posttranslationally modify substrates, and thereby alter the functions of their targets. The ubiquitination process is involved in various physiological responses, and dysregulation of components of the ubiquitin system has been linked to many diseases including skin cancer. The ubiquitin pathways activated among skin cancers are highly diverse and may reflect the various characteristics of the cancer type. Basal cell carcinoma and squamous cell carcinoma, the most common types of human skin cancer, are instances where the involvement of the deubiquitination enzyme CYLD has been recently highlighted. In basal cell carcinoma, the tumor suppressor protein CYLD is repressed at the transcriptional levels through hedgehog signaling pathway. Downregulation of CYLD in basal cell carcinoma was also shown to interfere with TrkC expression and signaling, thereby promoting cancer progression. By contrast, the level of CYLD is unchanged in squamous cell carcinoma, instead, catalytic inactivation of CYLD in the skin has been linked to the development of squamous cell carcinoma. This paper will focus on the current knowledge that links CYLD to nonmelanoma skin cancers and will explore recent insights regarding CYLD regulation of NF-**κ**B and hedgehog signaling during the development and progression of these types of human tumors.

## 1. The Ubiquitin System

**Figure 1:**
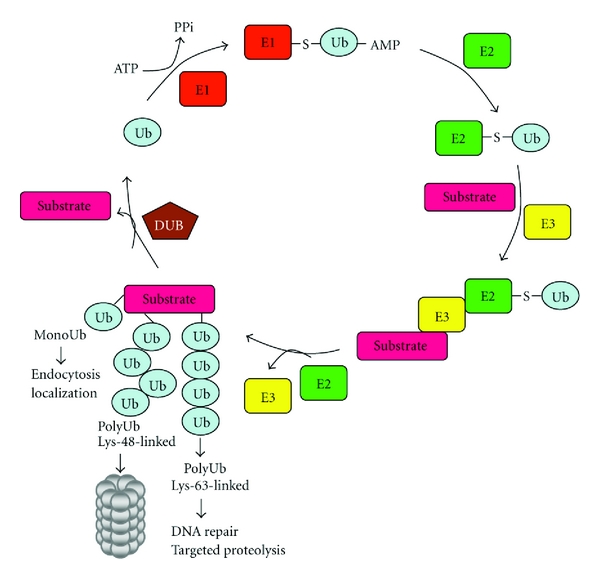
Ubiquitin conjugation pathway. The ubiquitin pathway relies on a cascade of three enzymes, named ubiquitin-activating enzyme (E1), ubiquitin-conjugating enzyme (E2), and ubiquitin-ligating enzyme (E3), which conjugate ubiquitin to target proteins. First, ubiquitin is activated by the E1 enzyme in an ATP-dependent manner. As a consequence, ubiquitin is covalently bound to E1 via thioester bond with cysteine residue in the active site of the E1 enzyme. The ubiquitin is transferred to the active cysteine in E2 enzyme. Finally, with the help of an E3 enzyme, ubiquitin is conjugated to its target substrate where different ubiquitin-ubiquitin linkages help to decide the fate of the modified protein. Ubiquitination is primarily associated with degradation of the tagged protein by the 26S proteasome (Lys-48), but it has also nondegradative functions (Lys-63) such as the regulation of DNA repair and endocytosis amongst other functions. Enzymes known as deubiquitinating enzymes (DUBs) can remove ubiquitin from proteins.

The posttranslational modification of different proteins via direct, covalent ubiquitin attachment regulates stability, function, and localization of the modified protein. Ubiquitin is covalently bound to *ε*-amino groups of lysine (K) residues or, less commonly, to amino termini of substrate proteins. Substrates can be modified, either by a monoubiquitin, or a polyubiquitin chain(s) (polyubiquitination). Monoubiquitination is involved in at least three distinct cellular functions: histone regulation, endocytosis, and the budding of retroviruses from the plasma membrane. Different forms of polyubiquitin are known to target proteins for different cellular fates. For example, K48-, K29-, and K11-linked polyubiquitin chains target substrates for proteasome-mediated degradation. K63-linked polyubiquitin chains appear to function independently of proteasomal targeting and instead trigger diverse cellular processes, including DNA repair, receptor trafficking, and endocytosis or modulate the activity of kinases and transcription factors, thereby enable cells to interpret signals from their environment [1, 2] (Figure 1). Ubiquitin modification is reversed by the action of enzymes known as deubiquitinating enzymes (DUBs) [3] such as cylindromatosis gene (CYLD). DUBs generally cleave mono- or polyubiquitin chains with substrate specificity, and, as has been established, DUBs can distinguish between the different types of linkages in the polyubiquitin chains. In this regard, CYLD was shown to only disassemble K63-linked polyubiquitin chains from different substrates. Recent molecular investigation has provided us with a better understanding of how the tumor suppressor gene CYLD can regulate a variety of signaling pathways that are linked to tumor cell proliferation and survival by cleavage of the ubiquitin chains from target proteins. This paper will focus on the role of CYLD in the nonmelanoma skin cancers, basal cell carcinoma (BCC), and squamous cell carcinoma (SCC). 

## 2. CYLD and Cylindromatosis

CYLD was originally discovered in families with multiple cylindromas, a rare benign skin disease. Linkage analysis mapped the susceptibility CYLD gene to a single locus on chromosome 16q in affected families, where both germline and somatic mutations occurred [4]. This discovery suggested that loss of heterozygosity (LOH) of this novel tumor suppressor gene due to mutations is associated with the development of inherited familial cylindromatosis [5].

Brooke-Spiegler syndrome (BSS, OMIM 605041), familial cylindromatosis (FC, OMIM 132700), and multiple familial trichoepithelioma (MFT, OMIM 601606) were originally described as separate diseases that display certain clinical similarities. Patients with BSS are predisposed to the progressive development of cylindromas, trichoepitheliomas, and spiradenomas, whereas FC is characterized by multiple cylindromas and MFT by trichoepithelioma as the only tumor type. The inherited occurrence of cylindromas and trichoepitheliomas was also found to be combined within a single genetic defect [6, 7]. CYLD function, however, is not limited to its role in the etiology of cylindromas and trichoepitheliomas but has also been shown to act as a tumor suppressor in multiple types of human cancers such as melanoma, colon, lung, and multiple myeloma (for paper, see [8]).

Human and mouse CYLD genes are very closely related and highly conserved (95% homology). The CYLD gene product is ubiquitously expressed in humans and mice. In the skin, specifically in hair follicles, CYLD levels were found to be elevated in the catagen phase of the hair cycle when cell proliferation ceases [9]. CYLD protein contains three cytoskeletal-associated protein-glycine-conserved (CAPGly) domains and one zinc-finger-like B-box motif within the ubiquitin carboxy-terminal hydrolases domain (UCH or USP; Ub-specific proteases). The CAP-Gly domains of CYLD are important for CYLD relocalization or binding to substrates and function as scaffolding [10], whereas the USP domain hydrolyzes ubiquitin chains [11]. In vitro and in vivo analyses have previously indicated that a single mutation in the Cys601 (CYLDC/S) located in the USP domain abolishes the deubiquitin activity of CYLD [12–14].

Recently, a number of specific substrates for CYLD were identified from which CYLD could remove the ubiquitin chains by direct association. Furthermore, insights into the normal functions and signaling interactions of the CYLD gene product indicate that CYLD interferes with NF-*κ*B-, JNK-, WNT-, and p38MAPK signaling to limit inflammation and cancer depending upon the tissue-/cell-type or in vivo model system [10]. TRAF-2 and TRAF-6 were the first specific CYLD substrates identified from which CYLD was shown to remove K63- linked polyubiquitin chains while also attenuating TNF-*α*-induced classical NF-*κ*B signaling [12–14].

## 3. Regulation of Signaling by Ubiquitination

### 3.1. NF-*κ*B Signaling

**Figure 2:**
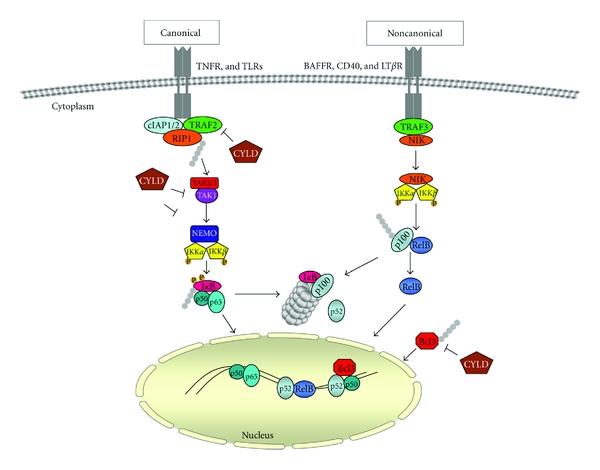
The role of CYLD in canonical and noncanonical NF-*κ*B pathway. In the canonical NF-*κ*B pathway, NF-*κ*B dimmers such as p65/p50 are maintained in the cytoplasm by interaction with an I*κ*B*α* protein. The binding of a ligand to a cell-surface receptor activates TAK1 which in turn activates an IKK complex, containing-*α*, -*β*, and NEMO, which is responsible for phosphorylation of IKK-*β*. IKK-*β* then phosphorylates I*κ*B-*α*, leading to K48-ubiquitination and degradation of this protein. p65/p50 then freely enters the nucleus to turn on target genes. The noncanonical pathway is largely for the activation of p100/RelB complexes and differs from the classical pathway in that only certain receptor signals, activate this pathway and it proceeds through an IKK complex that contains two IKK-*α* subunits but not NEMO. In the noncanonical NF-*κ*B pathway, receptor binding leads to activation of the NF-*κ*B-inducing kinase NIK, which phosphorylates and activates an IKK-*α* complex that in turn phosphorylates I*κ*B domain of p100, leading to its partial proteolysis and liberation of the p52/RelB complex. CYLD blocks canonical NF-*κ*B pathway by the removal of Lys-63 ubiquitinated chains from activated TRAFs, RIP, NEMO, and BCL3.

Nuclear factor kappa B (NF-*κ*B) is the generic name for a family of dimers formed by several proteins: NF-*κ*B1 (also known as p50/p105), NF-*κ*B2 (also known as p52/p100), c-Rel, RelA (also known as p65), and RelB. The different NF-*κ*B heterodimers recognize a common consensus DNA sequence and regulate a large number of target genes, especially those involved in the immune system and also genes involved in injury and stress [15]. In unstimulated cells, homodimers or heterodimers of the NF-*κ*B family members are bound to ankyrin-rich regions of inhibitor of NF-*κ*B (I*κ*B) proteins. This binding retains the dimers in the cytoplasm to prevent transcription of target genes. The NF-*κ*B1/p105 and NF-*κ*B2/p100 precursor proteins, which encode p50 and p52, also behave like I*κ*Bs, with the ankyrin repeats in their carboxyl-terminal. The I*κ*Bs and NF-*κ*B2/p100 are important targets of inducible regulatory pathways that mobilize active NF-*κ*B to the nucleus. NF-*κ*B signaling cascade results in the activation of classical/canonical or the alternative/noncanonical pathway depending on the type of stimuli (Figure 2). In general, growth factors and inflammatory mediators, hormones, and other signals can activate the classical NF-*κ*B pathway.

The well known that NF-*κ*B-dependent regulation of inflammatory response by TNF-*α* along the classical activation pathway includes recruitment and autoubiquitination of TRAFs through K63-linked ubiquitin chains. These ubiquitin chains act as molecular bridges that connect TRAF6 with TAB-TAK complex, which further induces IKK phosphorylation and activation (Figure 2). The latter phosphorylates I*κ*B-*α* become ubiquitinated and is rapidly degraded through proteasome. Thus, NF-*κ*B complexes (p65/p50) are freed and translocate to the nucleus for the expression of corresponding target genes (Figure 2) [16]. The alternative NF-*κ*B pathway depends on IKK*α* but not on IKK*β* or NEMO. The target for activated IKK*α* is the inhibitory ankyrin protein NF-*κ*B2/p100, which is phosphorylated by IKK*α* at its carboxyl terminus. Phosphorylation of NF-*κ*B2/p100 is followed by the ubiquitin-dependent degradation of p100 into the p52 product and nuclear translocation of p52 (Figure 2). Examples of stimuli that activate the alternative pathway include Lymphotoxin (LT)*β* R, B-cell activating factor receptor (BAFFR), receptor activator of NF-*κ*B (RANK), and CD40 [17].

There are several lines of evidence suggesting that dysregulation of the NF-*κ*B system may lead to gene expression patterns involved in tumor growth and invasion. In these studies, functional blockade of NF-*κ*B was achieved by targeting to skin expression a repressor mutant of I*κ*B-*α* inhibitory protein (I*κ*B*α*M). These studies could show spontaneous development of malignant SCC in transgenic mice expressing I*κ*B*α*M or the production of large neoplasms and SCC in human skin tissues by coexpression of I*κ*B*α*M and Ras [18, 19].

One known regulator of nuclear NF-*κ*B activity is BCL3, a protein closely related to I*κ*B. BCL3 was originally identified in a subgroup of B cell leukemias that carry a translocation of the immunoglobulin heavy chain (IgH) promoter adjacent to the BCL3 coding region [20]. Since p50 and p52 lack transactivation domains, BCL3 functions as a transcriptional coactivator through its association with p50 and p52 (Figure 2). In many different tumor types, BCL3 in association with p50 or p52 promotes oncogenesis through the activation of cyclin D1. Melanoma, hepatocellular carcinoma, and breast cancer are instances where cyclin D1 expression is upregulated due to the BCL3-p50 or BCL3-p52 association [21–23].

### 3.2. Hedgehog Signaling

The hedgehog (Hh) pathway is one of the fundamental signal transduction pathways in animal development, stem-cell maintenance, and carcinogenesis. The Hh gene was first discovered in Drosophila, and members of the family have since been found in most metazoans [24]. Hh proteins are synthesized as precursor proteins and comprise several different motifs and domains consisting of a signal peptide for protein export, a secreted N-terminal domain that acts as a signaling molecule, and an autocatalytic C-terminal domain [24].

**Figure 3:**
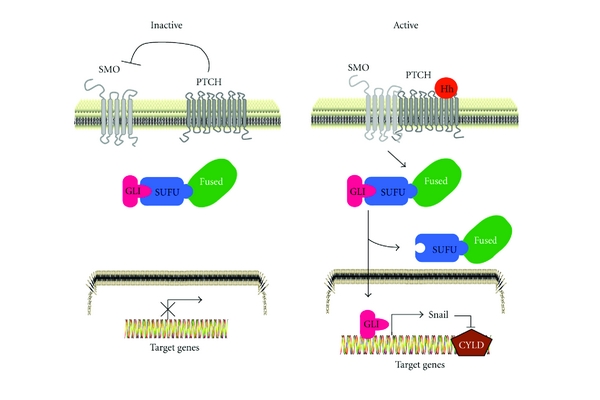
Downregulation of CYLD in BCC mediated by hedgehog signaling pathway. In the absence of ligand, the hedgehog (Hh) signaling pathway is inactive (left). Patched (PTCH) inhibits the activity of Smoothened (SMO), which in turn is unable to activate GLI transcription factors through interactions with FUSED and Suppressor of FUSED (SUFU). The binding of SUFU also prevents the transcription of Hh target genes. Binding of the Hh ligand inhibits PTCH and activates hedgehog pathway (right) through derepression of SMO and translocation of GlLI to the nucleus. Nuclear GLI activates target gene expression, including PTCH, GLI, and Snail. Expression of Snail leads to transcriptional inactivation of CYLD by recruitment of Snail to the CYLD promoter.

In general, mature Hh proteins secreted from producer cells induce effects on target cells expressing Hh receptor Patched (PTCH) [25]. PTCH indirectly transduces Hh signals to the intracellular signaling cascade through Smoothened (SMO) which is a typical seven-transmembrane receptor [26]. SMO activation is further mediated through phosphorylation of its carboxyl terminal intracellular tail by protein kinases including protein kinase A (PKA) and casein kinase I (CKI). In the presence of Hh signaling, SMO induces suppressor of fused (SUFU) inactivation and initiates stabilization and nuclear accumulation of GLI (glioma-associated oncogene) family members (GLI1, GLI2, and GLI3) [27, 28]. Similar to the role of I*κ*B-*α* in retaining NF-*κ*B in the cytoplasm in an inactive form, SUFU interacts with GLI in the cytoplasm to prevent its nuclear translocation and transactivation. However, cells treated with an activator of Hh signaling leads to the ubiquitin-mediated degradation of SUFU and release of GLI. Unbound GLI in the cytoplasm actively translocates into the nucleus, where it promotes the transcription of its target genes that are involved in different cellular processes (Figure 3). The GLI transcription factors can also inhibit transcription by binding to GLI responsive genes and by interacting with the transcription complex [29, 30]. In general, GLI1 seems to have only activator functions whereas GLI2 and GLI3 can be activators or suppressors of transcription in a context-dependent manner.

Alternative regulation of Hh signaling pathway takes place directly through ubiquitin-mediated degradation of GLI, which is facilitated by three different ubiquitin ligases: SCF*β*-Trcp, Cul3-HIB, and Itch [31]. In the absence of Hh, phosphorylation of GLI by PKA, glycogen synthase kinase 3 (GSK3), and CK1 triggers binding of GLI to SCF*β*-Trcp ubiquitin ligase complexes and consequent proteolysis. In addition to SCF*β*-Trcp-mediated degradation, GLI proteins are also subject to proteasome degradation through the action of Roadkill (Rdx) (also called HIB), which encodes a substrate specific receptor for the Cul3-based E3 ubiquitin ligase [32, 33]. Hh signaling induces HIB expression, which serves as a feedback control limiting GLI activity after pathway activation. Recently, it has been demonstrated that GLI signaling is suppressed by Numb, an evolutionarily-conserved developmental protein that has critical roles in cell differentiation. The suppression of GLI signaling involves the ubiquitin-regulated processing of GLI-mediated by Numb and the HECT domain E3 ubiquitin ligase Itch [34]. Thus, different E3 ubiquitin ligases have an impact on the Hh pathway at different levels to control the spatial and temporal activity of GLI proteins.

In addition to ubiquitination, several histone-modifying enzymes and chromatin remodeling proteins have been shown to influence the transcriptional activity of GLI proteins. GLI1 and GLI2 are modified by acetylation and class I histone deacetylases regulate their transcriptional activities [35]. Recent study has also identified Snf5, a core member of the ATP-dependent switch (SWI)/sucrose nonfermenting (SNF) chromatin remodeling complex, and several other SWI/SNF complex subunits as GLI1-specific interacting proteins. Moreover, inactivation of Snf5, which localizes to GLI1 promoter, resulted in the upregulation of GLI1 expression. This result indicates that the SWI/SNF complex is a regulator of Hh signaling by suppression of GLI1 transcriptional activity [36]. All three GLI proteins are targets of small ubiquitin-related modifier (SUMO) conjugation. Expression of the SUMO E3 ligase Pias1 promotes GLI transcriptional activity, suggesting that GLI protein sumoylation acts as a potential regulatory mechanism in Hh signaling [37].

The first link between the Hh pathway and BCC came from the discovery of loss-of-function mutations of PTCH1 gene in Gorlin syndrome [25, 38]. These patients are strongly predisposed to the development of BCCs at early age. It has been established that the majority of BCCs arise sporadically on sun exposed areas of the skin and BCCs routinely carry mutations in PTCH1 [39]. Mutations in several other genes encoding components of Hh signalling such as SMO, SUFU, and abnormalities of Hh pathway transcription factors GLI can also contribute to sporadic BCC development (for paper see [40]).

## 4. CYLD and Nonmelanoma Skin Cancer

### 4.1. CYLD and Basal Cell Carcinoma

CYLD is profoundly downregulated at both the mRNA and protein levels in different stages of BCC compared to keratinocytes in the normal epidermis [41]. Reduced expression of CYLD is a result of recruitment of the transcription repressor protein Snail to the CYLD promoter which leads to silencing of its expression [41] (Figure 3). Snail was identified as an early GLI1-responsive gene in the skin and has been shown to be coexpressed with GLI1 in skin tumors [42]. In keratinocytes, the forced expression of GLI1, or treatment with puromorphamine (previously found to activate the Hh pathway), elevated the levels of Snail and inhibited CYLD expression. In contrast, knockdown of Snail or treatment of mouse BCC cell lines with cyclopamine, which inhibits the Hh pathway, led to an increase in the levels of CYLD [41]. The consequences of the reduced levels of CYLD cause by GLI1-Snail1 signaling affected proliferation and cell cycle by promoting G1 to S phase progression. BCL3 was identified as a CYLD-specific substrate in BCC and treatment of BCC cell lines with cyclopamine rescued the expression of CYLD. CYLD in turn reduced the ubiquitination and nuclear localization of BCL3 [41]. In addition to BCCs, which arises in mice expressing GLI1 in the skin, these mice also develop cylindroma and trichoepitheliomas [43], suggesting that the loss or inactivation of CYLD in different skin cancer diseases can affect the same signaling pathway.

Molecular genetic analysis has demonstrated the aforementioned mutation at 9q22–31 in 70% of sporadic BCCs [44, 45]. However, there is no evidence in the literature thus far that indicates alterations on chromosome 16, where the CYLD gene is located. LOH on chromosome 16 was also investigated using three microsatellite markers (D16s407, D16s308, and D16s304) in the BCC originating from skin adnexal tumors [46]. Such tumors are an uncommon but related group of benign neoplasms that differentiate towards epidermal appendages rather than surface epidermis. Laser capture microdissection showed LOH in only one patient out of nine. Moreover, this LOH was identified in one of the markers, but not in the others, indicating that CYLD mutation has no role in the development of BCC originating from adnexal tumors [46]. Furthermore, analysis of two large nodular BCCs from a 46-year-old man with an almost 20-year history of multiple discrete and confluent papules and nodules on the face showed a germline substitution mutation (c.1684 + 1G > A) of the CYLD gene in the DNA isolated from tissue block and peripheral blood [47]. This previously uncharacterized mutation occurred in the exon-intron junction and could affect correct splicing [47]. However, analysis performed in eight tissue blocks containing malignant BCCs from the same patient identified CYLD gene LOH and sequence mutation without finding any correlation between the tumor type and somatic event [47]. Mutations in PTCH1, which activates Hh signaling, were originally identified in BCC patients [25, 48]. These mutations are thought to be the major cause of Hh pathway activation in BCCs. A recent study by Kazakov et al., analyzing peripheral blood and tumor tissues of a family having small nodular BCC arising from multiple trichoepitheliomas, found no mutations or LOH in CYLD or in PTCH [49].

The Trk family of neurotrophin receptors plays a critical role in the development and maintenance of the central and peripheral nervous systems. This homologous family of tyrosine kinase receptors consists of TrkA, TrkB, and TrkC. The primary ligand for these receptors is nerve growth factor (NGF) [50]. NGF stimulation of PC12 cells that are derived from transplantable rat pheochromocytoma promotes K63-mediated deubiquitination of TrkA by CYLD which further affects TrkA internalization and signaling [51, 52]. Aside from CYLD interfering with TrkA signaling, TrkB and TrkC are overexpressed in tumors lacking CYLD expression including cylindroma, spiradenoma, trichoepithelioma, and BCC [53]. The increased level of TrkC in BCC has been implicated in the proliferative advantage of cells, where constitutively active Hh signaling due to the loss of functional PTCH occurs. As it was shown that CYLD can interfere with Trk signaling [53], although it is not entirely clear how loss of CYLD can promote overexpression of TrkC, a block of TRK receptor signaling has been proposed to represent a novel therapeutic target in tumors with loss of CYLD function including BCC [53].

### 4.2. CYLD and Squomous Cell Carcinoma

In a large tissue microarray-based study consisting of 131 patients with SCC tumor specimens, the expression levels of CYLD were not altered [54]. Furthermore, skin tumors chemically induced by using DMBA/TPA in CYLD-deficient mice resulted in the development of skin papillomas without any signs of SCC [55]. Instead, two recent studies using transgenic animals were able to identify the role of CYLD mutation in the development of SCC [56, 57]. Genetically modified complete knock out versus knock-in mouse models could be the explanation for the differences in the phenotype.

**Figure 4:**
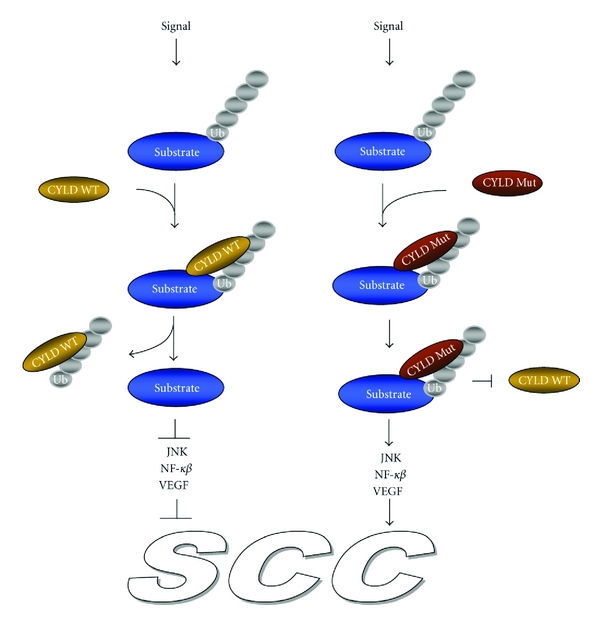
Catalytic inactive mutant form of CYLD promotes development of squamous cell carcinoma. Keratinocyte stimulation with DMBA, TPA, or UV-B causes ubiquitination-mediated signaling and activation of JNK, NF-*κ*B pathways, and the upregulation of VEGF gene expression. Wild-type CYLD (CYLD WT) by interaction and deubiquitination of its specific substrate blocks the propagation of downstream signaling pathways. The mutant form of CYLD (CYLD Mut), which binds to the substrate, is unable to remove K63-polyubiquitin chains from the target protein and blocks the downstream signaling pathways. In addition, the mutant form of CYLD competes with the wild-type CYLD for the same binding site on the substrate. Constitutive activation of JNK and NF-*κ*B signaling in CYLD mutant cells (CYLD Mut), leads to development of squamous cell carcinoma (SCC).

TPA or UV-B stimulation of CYLD-deficient keratinocytes causes K63 ubiquitination-mediated nuclear translocation of BCL3. In the nucleus, BCL3 together with p52 binds to the cyclin D1 promoter and increases proliferation of these cells. In contrast, wild-type keratinocytes have reduced proliferation rates compared to the knockout cells due to the interaction of BCL3 with CYLD in the cytoplasm, where removal of K63 ubiquitin chains by CYLD occurs [55]. Transgenic animals expressing the mutant form of CYLD (CYLDC/S) which are unable to act as DUBs convert epidermal cells into malignant cells. The tumor epidermal cells expressing CYLDC/S in addition to being unable to remove ubiquitin chains from IKK (a downstream effector in NF-*κ*B signaling) also contain an active form of p65. Moreover, these cells show an increase in the number of cells having nuclear localized BCL3 and p52 responsible for cyclin D1 expression [57]. In addition, epithelial cells from CYLDC/S mice favor the malignancy by enhancing cell proliferation, migration, survival, and angiogenesis. This phenotype is partially dependent on elevated levels of vascular endothelial growth factor (VEGF) (Figure 4). However, future studies are needed to establish downstream signaling for CYLDC/S-mediated changes in VEGF levels. It is known that VEGF overexpression in murine epidermis predisposes mice to tumor development [58] and very recently it has been reported that VEGF plays a significant role not only in the first stage of angiogenesis establishment, but also in the advanced stages of several malignant tumor progressions including nonmelanoma skin cancer [58].

In another relevant study, mice expressing keratin 14 promoter-driven CYLD catalytic inactive mutant (CYLDm, lacking the 21 amino acid residues at the C-terminal end) did not develop any spontaneous skin tumors [56]. Instead, exposure of newborn CYLDm mice to DMBA/TPA resulted in cell malignancy and metastasis in a JNK/AP1-dependent manner [56]. In this pathway, CYLDm increased the basal levels of c-Jun and c-Fos and sustained their activation status in response to EGF treatment in an ubiquitin- and proteasome-dependent manner [56]. The increased nondegradable c-Jun/c-Fos protein was suggested as being responsible for malignant and metastatic tumor development in cancers associated with loss of functionmutations in the CYLD gene (Figure 4) [56].

## 5. Summary

We have surveyed the role of the deubiquitination enzyme CYLD in nonmelanoma skin cancers (BCCs and SCCs). BCCs differ from SCCs in many respects. While BCCs develop de novo, formation of skin SCC is a multiple-step process. BCCs exhibit a relatively simple genotype with few aberrations and show themselves to be highly independent of the immune system. Further, PTCH and SMO gene mutations are important in the development of BCCs [59]. On the other hand, SCCs develop from precursor lesions and accumulate a highly complex genotype. The great number of chromosomal aberrations, for example, loss of 3p, loss of 9p, and gain of 3q, 9q, and 11q as well as gene mutations such as p53, p16^INK4^, Ras, c-myc, and EGFR, has been related to SCC development [60, 61].

In BCC, suppression of the CYLD gene product is caused by the transcriptional regulator Snail, whose expression is activated by GLI1. Since Downregulation or even loss of CYLD expression could be observed in the early phase of BCC samples with different degrees of invasive behavior and tumor size, this suggests that CYLD repression occurs in an early stage of BCC development. BCC is the most common form of cancer in humans and is believed to arise from hair follicles. Since Snail and GLI1 transcripts are elevated in normal anagen (growth) phase of hair follicles, in contrast to CYLD whose expression is elevated only during the catagen (regression) phase of the hair cycle, one might hypothesize that disruption of expression of these genes may initiate growth of BCC. Downregulation of CYLD in BCC was also shown to interfere with TrkC expression and signaling, which under normal conditions is expressed in hair follicles. It is highly probable that elevated TrkC expression promotes the proliferative advantage of cells in cases where constitutively active Hh signaling following loss of functional PTCH occurs.

As the level of CYLD is unchanged in SCC compared to epidermal keratinocytes, catalytic inactivation of CYLD in skin has been connected to the conversion of epidermal cells to malignant cells. SCC derived from CYLD mutant cells shows an increase in the nuclear localization of BCL3 and p52 as well as an increase in cyclin D1 expression. In addition, VEGF expression, which is a well-known growth factor that stimulates angiogenesis and metastasis, was also elevated. The role of VEGF in the advanced stages of tumor progression in nonmelanoma skin cancer has recently been identified [58].

In general, ubiquitination-dependent degradation plays a major role in the progression of skin cancers. Recently, the proteasome inhibitor Bortezomib (Velcade or PS-341), the first therapeutic proteasome inhibitor for treating relapsed multiple myeloma, was shown to suppress the growth of SCC cell lines by inhibiting the NF-*κ*B pathway and VEGF levels [62]. Furthermore, cisplatin or cetuximab also enhanced the effect of bortezomib in SCC cell lines [63, 64]. It is not clear thus far whether Bortezomib has any effect on BCCs, but the inhibition of Hh signaling is frequently employed as a useful treatment for this type of cancer. Cyclopamine, GDC-0449, and LDE-225 are a few examples of compounds that are selective Hh inhibitors and are currently under investigation in ongoing trials.

Previous studies have identified correlations in the alteration of Hh and Wnt pathways in BCCs [65–69] and recently a population of cancer stem cells in mouse early SCCs demonstrating dependence on *β*-catenin (Wnt/*β*-catenin signaling) activation. In human cylindroma, CYLD negatively regulates Wnt/*β*-catenin signaling via deubiquitination of Dishevelled, which is a key component in Wnt-mediated *β*-catenin nuclear translocation. Further studies need to establish whether CYLD regulation of Wnt signaling has a direct effect on the development of BCCs and SCCs.

## 6. Conclusions and Future Directions

The human genome encodes a large number of ubiquitin ligases and DUBs involved in the regulation of different pathways. The challenge in this field is to find the signaling pathways for ubiquitin ligases and DUBs that are specifically linked to cancer etiology. CYLD mutations or lack of CYLD expression has been connected with the development or progression of different types of skin cancer. In order to arrive at novel molecular strategies for different skin cancer prevention, future work needs to focus on the signaling pathway in defined target cell populations. This is also important, since BCC, which very rarely metastasizes, is believed to arise from hair follicles, whereas SCC, which has a greater metastatic potential, arises from the interfollicular epidermis. Moreover, loss of CYLD expression leads to BCC while mutation/s leading to catalytic inactivation of CYLD produces SCC. Such differences may explain why the function and substrate of CYLD differ depending on the cell of origin and tumor type.

## References

[1] Weissman AM (2001). Themes and variations on ubiquitylation. *Nature Reviews Molecular Cell Biology*.

[2] Pickart CM (2001). Mechanisms underlying ubiquitination. *Annual Review of Biochemistry*.

[3] Reyes-Turcu FE, Ventii KH, Wilkinson KD (2009). Regulation and cellular roles of ubiquitin-specific deubiquitinating enzymes. *Annual Review of Biochemistry*.

[4] Biggs PJ, Wooster R, Ford D (1995). Familial cylindromatosis (turban tumour syndrome) gene localised to chromosome 16q12-q13: evidence for its role as a tumour suppressor gene. *Nature Genetics*.

[5] Bignell GR, Warren W, Seal S (2000). Identification of the familial cylindromatosis tumour-suppressor gene. *Nature Genetics*.

[6] Welch JP, Wells RS, Kerr CB (1968). Ancell-Spiegler cylindromas (turban tumours) and Brooke-Fordyce Trichoepitheliomas: evidence for a single genetic entity. *Journal of Medical Genetics*.

[7] Bowen S, Gill M, Lee DA (2005). Mutations in the CYLD gene in Brooke-Spiegler syndrome, familial cylindromatosis, and multiple familial trichoepithelioma: lack of genotype-phenotype correlation. *Journal of Investigative Dermatology*.

[8] Massoumi R (2011). CYLD: a deubiquitination enzyme with multiple roles in cancer. *Future Oncology*.

[9] Moriwaki K, Tsuruta D, Sugawara K, Kobayashi H, Massoumi R, Ishii M (2007). A role of CYLD in hair cycling mouse. *Journal of Investigative Dermatology*.

[10] Massoumi R (2010). Ubiquitin chain cleavage: CYLD at work. *Trends in Biochemical Sciences*.

[11] Komander D, Lord CJ, Scheel H (2008). The structure of the CYLD USP domain explains its specificity for Lys63-linked polyubiquitin and reveals a B box module. *Molecular Cell*.

[12] Brummelkamp TR, Nijman SMB, Dirac AMG, Bernards R (2003). Loss of the cylindromatosis tumour suppressor inhibits apoptosis by activating NF-*κ*B. *Nature*.

[13] Trompouki E, Hatzivassillou E, Tsichritzis T, Farmer H, Ashworth A, Mosialos G (2003). CYLD is a deubiquitinating enzyme that negatively regulates NF-*κ*B activation by TNFR family members. *Nature*.

[14] Kovalenko A, Chable-Bessia C, Cantarella G, Israël A, Wallach D, Courtois G (2003). The tumour suppressor CYLD negatively regulates NF-*κ*B signalling by deubiquitination. *Nature*.

[15] Bonizzi G, Bebien M, Otero DC (2004). Activation of IKK*α* target genes depends on recognition of specific *κ*B binding sites by RelB:p52 dimers. *EMBO Journal*.

[16] Ghosh S, Karin M (2002). Missing pieces in the NF-*κ*B puzzle. *Cell*.

[17] Shen RR, Hahn WC (2011). Emerging roles for the non-canonical IKKs in cancer. *Oncogene*.

[18] Van Hogerlinden M, Rozell BL, Ährlund-Richter L, Toftgård R (1999). Squamous cell carcinomas and increased apoptosis in skin with inhibited Rel/nuclear factor-*κ*B signaling. *Cancer Research*.

[19] Dajee M, Lazarov M, Zhang JY (2003). NF-*κ*B blockade and oncogenic Ras trigger invasive human epidermal neoplasia. *Nature*.

[20] McKeithan TW, Ohno H, Diaz MO (1990). Identification of a transcriptional unit adjacent to the breakpoint in the 14;19 translocation of chronic lymphocytic leukemia. *Genes Chromosomes and Cancer*.

[21] Cogswell PC, Guttridge DC, Funkhouser WK, Baldwin AS (2000). Selective activation of NF-*κ*B subunits in human breast cancer: potential roles for NF-*κ*B2/p52 and for Bcl-3. *Oncogene*.

[22] Massoumi R, Kuphal S, Hellerbrand C (2009). Down-regulation of CYLD expression by Snail promotes tumor progression in malignant melanoma. *Journal of Experimental Medicine*.

[23] Park SG, Chung C, Kang H, Kim JY, Jung G (2006). Up-regulation of cyclin D1 by HBx is mediated by NF-*κ*B2/BCL3 complex through *κ*B site of cyclin D1 promoter. *Journal of Biological Chemistry*.

[24] Bürglin TR (2008). The Hedgehog protein family. *Genome biology*.

[25] Johnson RL, Rothman AL, Xie J (1996). Human homolog of patched, a candidate gene for the basal cell nevus syndrome. *Science*.

[26] Van den Heuvel M, Ingham PW (1996). Smoothened encodes a receptor-like serpentine protein required for hedgehog signalling. *Nature*.

[27] Katoh M, Hirai M, Sugimura T, Terada M (1995). Cloning and characterization of MST, a novel (putative) serine/threonine kinase with SH3 domain. *Oncogene*.

[28] Varjosalo M, Björklund M, Cheng F (2008). Application of active and kinase-deficient kinome collection for identification of kinases regulating Hedgehog signaling. *Cell*.

[29] Kinzler KW, Bigner SH, Bigner DD (1987). Identification of an amplified, highly expressed gene in a human glioma. *Science*.

[30] Ruiz i Altaba A, Mas C, Stecca B (2007). The Gli code: an information nexus regulating cell fate, stemness and cancer. *Trends in Cell Biology*.

[31] Jiang J (2006). Regulation of Hh/Gli signaling by dual ubiquitin pathways. *Cell Cycle*.

[32] Hooper JE, Kent D, Bush EW (2006). Roadkill attenuates Hedgehog responses through degradation of Cubitus interruptus. *Development*.

[33] Jiang J, Zhang Q, Zhang L, Wang B, Ou CY, Chien CT (2006). A Hedgehog-induced BTB protein modulates Hedgehog signaling by degrading Ci/Gli transcription factor. *Developmental Cell*.

[34] Gulino A, Di Marcotullio L, Ferretti E (2006). Numb is a suppressor of Hedgehog signalling and targets Gli1 for Itch-dependent ubiquitination. *Nature Cell Biology*.

[35] Gulino A, Canettieri G, Di Marcotullio L (2010). Histone deacetylase and Cullin3-REN KCTD11 ubiquitin ligase interplay regulates Hedgehog signalling through Gli acetylation. *Nature Cell Biology*.

[36] Dorsch M, Jagani Z, Mora-Blanco EL (2010). Loss of the tumor suppressor Snf5 leads to aberrant activation of the Hedgehog-Gli pathway. *Nature Medicine*.

[37] Cox B, Briscoe J, Ulloa F (2010). SUMOylation by pias1 regulates the activity of the hedgehog dependent gli transcription factors. *PLoS ONE*.

[38] Hahn H, Wicking C, Zaphiropoulos PG (1996). Mutations of the human homolog of drosophila patched in the nevoid basal cell carcinoma syndrome. *Cell*.

[39] Gailani MR, Stahle-Backdahl M, Leffell DJ (1996). The role of the human homologue of Drosophila patched in sporadic basal cell carcinomas. *Nature Genetics*.

[40] Li C, Chi S, Xie J (2011). Hedgehog signaling in skin cancers. *Cellular Signalling*.

[41] Kuphal S, Shaw-Hallgren G, Eberl M (2011). GLI1-dependent transcriptional repression of CYLD in basal cell carcinoma. *Oncogene*.

[42] Li X, Deng W, Nail CD (2006). Snail induction is an early response to Gli1 that determines the efficiency of epithelial transformation. *Oncogene*.

[43] Nilsson M, Undèn AB, Krause D (2000). Induction of basal cell carcinomas and trichoepitheliomas in mice overexpressing GLI-1. *Proceedings of the National Academy of Sciences of the United States of America*.

[44] Gailani MR, Bale SJ, Leffell DJ (1992). Developmental defects in Gorlin syndrome related to a putative tumor suppressor gene on chromosome 9. *Cell*.

[45] Quinn AG, Sikkink S, Rees JL (1994). Delineation of two distinct deleted regions on chromosome 9 in human non- melanoma skin cancers. *Genes Chromosomes and Cancer*.

[46] Leonard N, Chaggar R, Jones C, Takahashi M, Nikitopoulou A, Lakhani SR (2001). Loss of heterozygosity at cylindromatosis gene locus, CYLD, in sporadic skin adnexal tumours. *Journal of Clinical Pathology*.

[47] Kazakov DV, Schaller J, Vanecek T, Kacerovska D, Michal M (2010). Brooke-Spiegler syndrome: report of a case with a novel mutation in the CYLD gene and different types of somatic mutations in benign and malignant tumors. *Journal of Cutaneous Pathology*.

[48] Wolter M, Reifenberger J, Sommer C, Ruzicka T, Reifenberger G (1997). Mutations in the human homologue of the Drosophila segment polarity gene patched (PTCH) in sporadic basal cell carcinomas of the skin and primitive neuroectodermal tumors of the central nervous system. *Cancer Research*.

[49] Kazakov DV, Vanecek T, Nemcova J (2009). Spectrum of tumors with follicular differentiation in a patient with the clinical phenotype of multiple familial trichoepitheliomas: a clinicopathological and molecular biological study, including analysis of the CYLD and PTCH genes. *American Journal of Dermatopathology*.

[50] Brodeur GM, Minturn JE, Ho R (2009). Trk receptor expression and inhibition in neuroblastomas. *Clinical Cancer Research*.

[51] Wooten MW, Geetha T, Babu JR (2008). Essential role of sequestosome 1/p62 in regulating accumulation of Lys63-ubiquitinated proteins. *Journal of Biological Chemistry*.

[52] Geetha T, Jiang J, Wooten MW (2005). Lysine 63 polyubiquitination of the nerve growth factor receptor TrkA directs internalization and signaling. *Molecular Cell*.

[53] Rajan N, Elliott R, Clewes O (2011). Dysregulated TRK signalling is a therapeutic target in CYLD defective tumours. *Oncogene*.

[54] Vasiljević N, Andersson K, Bjelkenkrantz K (2009). The Bcl-xL inhibitor of apoptosis is preferentially expressed in cutaneous squamous cell carcinoma compared with that in keratoacanthoma. *International Journal of Cancer*.

[55] Massoumi R, Chmielarska K, Hennecke K, Pfeifer A, Fässler R (2006). Cyld inhibits tumor cell proliferation by blocking Bcl-3-dependent NF-*κ*B signaling. *Cell*.

[56] De Marval PM, Lutfeali S, Jin JY, Leshin B, Angelica Selim M, Zhang JY (2011). CYLD inhibits tumorigenesis and metastasis by blocking JNK/AP1 signaling at multiple levels. *Cancer Prevention Research*.

[57] Alameda JP, Moreno-Maldonado R, Navarro M (2010). An inactivating CYLD mutation promotes skin tumor progression by conferring enhanced proliferative, survival and angiogenic properties to epidermal cancer cells. *Oncogene*.

[58] Mirones I, Conti CJ, Martínez J, Garcia M, Larcher F (2009). Complexity of VEGF responses in skin carcinogenesis revealed through ex vivo assays based on a VEGF-A null mouse keratinocyte cell line. *Journal of Investigative Dermatology*.

[59] Athar M, Tang X, Lee JL, Kopelovich L, Kim AL (2006). Hedgehog signalling in skin development and cancer. *Experimental Dermatology*.

[60] Boukamp P (2005). Non-melanoma skin cancer: what drives tumor development and progression?. *Carcinogenesis*.

[61] Xie J (2008). Molecular biology of basal and squamous cell carcinomas. *Sunlight, Vitamin D and Skin Cancer*.

[62] Sunwoo JB, Chen Z, Dong G (2001). Novel proteasome inhibitor PS-341 inhibits activation of nuclear factor-*κ*B, cell survival, tumor growth, and angiogenesis in squamous cell carcinoma. *Clinical Cancer Research*.

[63] Fribley AM, Evenchik B, Zeng Q (2006). Proteasome inhibitor PS-341 induces apoptosis in cisplatin-resistant squamous cell carcinoma cells by induction of Noxa. *Journal of Biological Chemistry*.

[64] Wagenblast J, Baghi M, Arnoldner C (2009). Cetuximab enhances the efficacy of bortezomib in squamous cell carcinoma cell lines. *Journal of Cancer Research and Clinical Oncology*.

[65] Altaba AR, Mullor JL, Dahmane N, Sun T (2001). Wnt signals are targets and mediators of Gli function. *Current Biology*.

[66] Yamazaki F, Aragane Y, Kawada A, Tezuka T (2001). Immunohistochemical detection for nuclear *β*-catenin in sporadic basal cell carcinoma. *British Journal of Dermatology*.

[67] El-Bahrawy M, El-Masry N, Alison M, Poulsom R, Fallowfield M (2003). Expression of *β*-catenin in basal cell carcinoma. *British Journal of Dermatology*.

[68] Saldanha G, Ghura V, Potter L, Fletcher A (2004). Nuclear *β*-catenin in basal cell carcinoma correlates with increased proliferation. *British Journal of Dermatology*.

[69] Dlugosz AA, Yang SH, Andl T (2008). Pathological responses to oncogenic Hedgehog signaling in skin are dependent on canonical Wnt/*β*-catenin signaling. *Nature Genetics*.

